# Integrating pharmacogenetic and clinical factors to predict the C0/D/W-based tacrolimus phenotype in kidney transplantation

**DOI:** 10.3389/fphar.2026.1772820

**Published:** 2026-05-15

**Authors:** Andrea Guzmán-de Antonio, Carlos Jiménez-Martín, Elia B. Márquez-Cabello, Marcos Navares-Gómez, María O. López-Oliva, Elena González-García, Lucía Díaz-García, Rocío Rosas-Alonso, Irene García-García, Alberto M. Borobia, Antonio J. Carcas

**Affiliations:** 1 Clinical Pharmacology Department, La Paz University Hospital, IdiPAZ (Hospital La Paz Institute for Health Research), Faculty of Medicine, Universidad Autónoma de Madrid, Madrid, Spain; 2 Pharmacogenetics Laboratory, Genetics Department, La Paz University Hospital, Madrid, Spain; 3 Nephrology Department, La Paz University Hospital, Madrid, Spain; 4 Clinical Pharmacology Department, Hospital Universitario La Princesa, Instituto de Investigación Sanitaria La Princesa (IIS-Princesa), Madrid, Spain; 5 Experimental Therapies and Novel Biomarkers in Cancer, Hospital La Paz Institute for Health Research-IdiPAZ, Madrid, Spain

**Keywords:** C0/D/W ratio, CYP3A5, kidney transplantation, pharmacogenetics, tacrolimus

## Abstract

**Background:**

Tacrolimus shows substantial interindividual pharmacokinetic variability, complicating dose individualization in renal transplantation. The tacrolimus trough concentration-to-dose-to-weight ratio (C0/D/W) has been proposed as a simple surrogate of tacrolimus bioavailability, yet the extent to which pharmacogenetic and clinical factors explain this phenotype in stable adult kidney transplant recipients remains unclear.

**Methods:**

We conducted a single-centre retrospective study including 77 adult renal transplant recipients treated with prolonged-release tacrolimus formulations (Advagraf® or Envarsus®) and clinically stable. Tacrolimus exposure phenotype was defined using C0/D/W tertiles: the lowest tertile was classified as the high-metabolism phenotype and the highest tertile as the low-metabolism phenotype. Genotyping was performed using a customized OpenArray panel. The predictive performance of CYP3A5 rs776746 alone was assessed using diagnostic accuracy metrics. Multivariable logistic regression models integrating clinical covariates and genotype-based exposure scores were developed. Model performance was evaluated using McFadden’s pseudo-R^2^ and Receiver Operating Characteristic analysis with bootstrap internal validation.

**Results:**

The high- and low-metabolism groups comprised 39 and 38 patients, respectively. CYP3A5 expresser genotypes (*1 carriers) identified high-metabolism phenotype with a sensitivity of 48.7% and a specificity of 89.5%, yielding an overall accuracy of 68.8%. In multivariable analysis, CYP3A5 rs776746 score, age, and post-transplant period were associated with metabolism phenotype. The primary parsimonious model demonstrated good discrimination (apparent AUC 0.83; 95% CI 0.73–0.92), with an optimism-corrected AUC of 0.80 and a McFadden pseudo-R^2^ of 0.26. Exploratory analyses including additional pharmacogenetic variants such as ABCB1 rs1045642 provided only marginal improvement in model fit and did not justify increased model complexity.

**Conclusion:**

CYP3A5 genotype alone provides high specificity but limited sensitivity for predicting tacrolimus C0/D/W-based metabolism phenotype. Incorporation of key clinical variables, including age and time since transplantation, improves phenotype discrimination and provides a more comprehensive characterization of tacrolimus exposure variability. Additional pharmacogenetic markers showed limited incremental value. These findings support a combined genetic–clinical approach for tacrolimus dose individualization, although external validation in larger cohorts is warranted.

## 1 Introduction

Renal transplantation is widely regarded as the gold standard therapy for eligible patients with end-stage renal disease. Over the past decades, advances in immunosuppressive therapy have substantially improved medium- and long-term graft survival (Stratta et al., 2012; Khan et al., 2025). However, chronic immunosuppression continues to pose major clinical challenges, including graft dysfunction and rejection, susceptibility to viral infections, and adverse drug-related effects such as nephrotoxicity, diabetes, and hypertension. Optimizing immunosuppressive regimens to balance efficacy and safety therefore remains a central goal in transplant medicine (Jindal et al., 1997; de Mattos et al., 2000; Hoorn et al., 2011; Schütte-Nütgen et al., 2019).

Among immunosuppressive agents, tacrolimus is a cornerstone of post-transplant therapy. Its mechanism of action involves inhibition of the phosphatase calcineurin, thereby preventing dephosphorylation and nuclear translocation of the transcription factor NFAT, a critical step required for the transcription of IL-2–dependent genes (Kapturczak et al., 2004; Barbarino et al., 2013). Tacrolimus is a highly lipophilic drug with markedly variable bioavailability; on average, only about 25% of the administered dose is systemically available (Yu et al., 2018). Its metabolism occurs primarily in hepatic microsomes via cytochrome P450 enzymes, predominantly the CYP3A4 and CYP3A5 isoforms. In addition, it is a substrate of P-glycoprotein, a member of the ATP-Binding Cassette (ABC) transporter family (Barbarino et al., 2013; Yu et al., 2018).

Tacrolimus pharmacokinetics is substantially influenced by genetic polymorphisms in drug-metabolizing enzymes. According to the Clinical Pharmacogenetics Implementation Consortium (CPIC) guidelines, CYP3A5 is the main isoform affecting tacrolimus bioavailability. The presence of two *1 alleles (CYP3A5*1/*1) or one *1 and one *3 allele (CYP3A5*1/3) confer an “expresser” phenotype, associated with a normal metabolic capacity for the drug. Conversely, the presence of two mutated alleles (CYP3A5*3/*3) results in a non-expresser phenotype, leading to loss of enzymatic function and increased systemic exposure, and is often described as a slow-metabolizer status (Birdwell et al., 2015). However, because the *3 allele is highly prevalent in Caucasian populations, patients with this genotype are generally considered “normal metabolizers” for practical dosing purposes. In addition to CYP3A5*3, other clinically relevant variants such as CYP3A5*6, CYP3A5*7, and CYP3A4*22 have been described and are classified as Tier 1 alleles according to a joint consensus recommendation from the Association for Molecular Pathology, CPIC, College of American Pathologists, Dutch Pharmacogenetics Working Group, European Society for Pharmacogenomics and Personalized Therapy, and PharmGKB (Pratt et al., 2023). Other studies have also suggested that additional variants in the CYP3A4 and ABCB1 genes may contribute to variability in tacrolimus pharmacokinetics (Li et al., 2014; Kravljaca et al., 2016; Oetting et al., 2018). Beyond genetic factors, several non-modifiable variables, including age, sex, nutritional status, concomitant medications, and environmental influences, also affect tacrolimus pharmacokinetics. Moreover, additional clinical and therapeutic variables such as hematocrit, serum albumin, and corticosteroid dose may contribute to interindividual variability (Stratta et al., 2012).

The narrow therapeutic range and extensive inter- and intra-individual variability in tacrolimus blood concentrations make therapeutic drug monitoring (TDM) and careful dose adjustment indispensable. Tacrolimus also displays significant variability in drug response and adverse effects, underscoring the need for improved strategies to optimize its clinical use (Andrews et al., 2017).

One approach commonly used in clinical practice to support dose adjustment is the concentration-dose ratio (C0/D). This metric has been evaluated as a simple surrogate index of tacrolimus bioavailability and pharmacokinetics. The C0/D ratio tends to be highly variable during the first months after transplantation and stabilizes after approximately 6 months. Patients with lower C0/D values are considered to have a “rapid metabolizer” phenotype, whereas those with higher values are considered to have a “slow metabolizer” phenotype. Low C0/D values have been associated with decreased renal function, higher incidence of biopsy-proven rejection, and more frequent switches to alternative immunosuppressive regimens compared with patients presenting higher ratios (Thölking et al., 2014; 2019; Schütte-Nütgen et al., 2019; Thongprayoon et al., 2020). Some studies have already demonstrated an association between pharmacokinetic characterization using this ratio and pharmacogenetic polymorphisms (Van Gelder et al., 2020; Vidal-Alabró et al., 2024; Dung et al., 2025).

The combined analysis of genetic variants and the C0/D-based pharmacokinetic phenotype has been proposed as a complementary strategy to support individualized tacrolimus dose optimization in transplant recipients. However, the extent to which pharmacogenetic markers together with readily available clinical variables can explain the tacrolimus pharmacokinetic phenotype in stable adult renal transplant recipients remains insufficiently defined. In this study, we aimed (i) to evaluate the ability of the CYP3A5 genotype to predict the tacrolimus metabolism phenotype defined using a body weight–adjusted concentration-dose ratio (C0/D/W), and (ii) to develop a multivariable model integrating genetic and clinical determinants to improve phenotype discrimination.

## 2 Methods

### 2.1 Study design and genotyping

#### 2.1.1 Study population

This single-centre retrospective observational study was conducted in accordance with applicable legal requirements, the principles of the Declaration of Helsinki, and Good Clinical Practice (GCP) guidelines. The study protocol was reviewed and approved by the Ethics Committee of La Paz University Hospital (Madrid, Spain). A total of 77 renal transplant recipients were enrolled after providing written informed consent. Eligible participants were adults of either sex who were receiving tacrolimus in prolonged-release formulations (Advagraf® or Envarsus®) as their primary immunosuppressant and were clinically stable for at least 6 months post-transplant. Additional inclusion criteria included the ability to understand and comply with study procedures. Participants were allowed to withdraw at any time without consequences.

#### 2.1.2 Phenotypic classification

Tacrolimus exposure was quantified using the dose- and weight-normalized trough concentration (C0/D/W), obtained at the clinical visit immediately preceding informed consent. In this study, the tacrolimus metabolism phenotype was operationalized using the C0/D/W ratio, which serves as a pragmatic pharmacokinetic exposure marker integrating both metabolic capacity and formulation-dependent bioavailability. To enhance discrimination between metabolic phenotypes, only patients belonging to the lowest and highest tertiles of C0/D/W within the overall cohort were included in the analysis. The first tertile (C0/D/W < 0.01839 (ng.kg)/(mL.mg)) was defined as the high-metabolism phenotype, whereas the third tertile (C0/D/W > 0.03487 (ng.kg)/(mL.mg)) was defined as the low-metabolism phenotype. Individuals in both groups were subsequently matched by age and sex to enhance comparability. Clinical laboratory parameters and tacrolimus pharmacokinetic data were collected and managed using REDCap electronic data-capture tools.

#### 2.1.3 Sample collection and genotyping

Peripheral blood samples were collected at the time of the inclusion visit in 1.5 mL EDTA polypropylene tubes, aliquoted, and stored at −20 °C until further processing. Genomic DNA was extracted using the MagMAX™ DNA Multi-Sample Ultra Kit (Thermo Fisher Scientific, United States) on the KingFisher™ Apex System.

Genotyping was performed using a QuantStudio 12 K Flex qPCR instrument equipped with an OpenArray thermal block (Applied Biosystems, Thermo Fisher Scientific, United States) and a customized array. Candidate genes and SNPs were selected based on their functional relevance and previously reported associations with tacrolimus pharmacokinetics. Genotyping completeness exceeded 98% for all polymorphisms. A limited number of genotypes were classified as undetermined due to probe-hybridization failures or other technical limitations of the array; these samples were excluded from genotype-frequency and multivariable analyses.

### 2.2 Statistical analysis

#### 2.2.1 Baseline characteristics

Baseline demographic, clinical, pharmacokinetic characteristics were first summarized according to tacrolimus metabolism phenotype. Continuous variables were expressed as mean ± standard deviation (SD) or median and interquartile range (IQR), depending on distribution, and compared using Student’s t*-*test or Mann–Whitney *U* test. Categorical variables were reported as counts and percentages and compared using the chi-square test or Fisher’s exact test. Statistical significance was set at *p* < 0.05.

#### 2.2.2 CYP3A5 genotyping and phenotype prediction

To quantify the ability of CYP3A5 genotype alone to discriminate between high- and low-metabolism phenotypes, a 2 × 2 contingency table was constructed using CYP3A5 expressers (*1 carriers) versus non-expressers (*3/*3) and C0/D/W-based phenotype. Sensitivity, specificity, positive predictive value (PPV), negative predictive value (NPV), and overall accuracy were calculated.

#### 2.2.3 Genetic scoring and multicollinearity assessment

Candidate polymorphisms were selected *a priori* from the literature based on their documented functional relevance to tacrolimus pharmacokinetics, including variants classified as Tier 1 by current pharmacogenetic guidelines, as well as additional polymorphisms in CYP3A4 and ABCB1. Variants lacking variability or showing extremely low variability in the study population were excluded from the association analyses, whereas those with sufficient variability were retained for analysis. To determine the optimal genetic coding scheme, additive, dominant, and recessive inheritance models were explored for each polymorphism using linear regression with the C0/D/W ratio as the outcome. The additive model was selected for all variants as it provided the best balance of parsimony and fit (Akaike Information Criterion, ΔAIC <2). Accordingly, a genotype-based exposure score was constructed by assigning numeric weights to each polymorphism according to its known or expected effect on tacrolimus exposure. For each locus, homozygosity for the allele associated with increased tacrolimus exposure was assigned a value of 2 points, heterozygosity a value of 1 point, and homozygosity for the allele associated with lower exposure was assigned a value of 0 points. The specific variants considered included CYP3A5 rs776746, CYP3A4 rs2242480, and ABCB1 rs1045642, rs1128503, and rs2032582. Hardy-Weinberg equilibrium was assessed for all selected SNPs. Variants showing no significant deviation from HWE (p > 0.05) were considered to be in equilibrium.

To reduce redundancy due to linkage disequilibrium, multicollinearity among genetic scores was quantified using the Variance Inflation Factor (VIF), with values >4 considered indicative of moderate to relevant collinearity. In the presence of highly correlated variables within the same gene a conservative approach was applied to retain only the most representative polymorphism for subsequent multivariable modeling.

#### 2.2.4 Multivariable logistic regression

Multivariable logistic regression models were developed to evaluate clinical and pharmacogenetic determinants of tacrolimus metabolizer phenotypes. Clinical covariates were considered based on biological plausibility and exploratory univariable analyses, prioritizing variables with relevant associations. Formulation type was retained *a priori* due to its established influence on tacrolimus bioavailability. Variables derived from tacrolimus exposure, such as dose, trough concentration, and dose-adjusted indices were excluded to avoid circularity with the outcome. Dosing interval was omitted due to a highly imbalanced distribution across groups, resulted in quasi-complete separation and unstable coefficient estimates. Building upon these criteria, a parsimonious main model was first specified as a baseline for phenotype prediction. This model incorporated age, post-transplant period, and formulation type as key clinical factors, alongside the CYP3A5 rs776746 score as the primary genetic determinant. This framework was then extended by incorporating additional pharmacogenetic variants (CYP3A4 and ABCB1 polymorphisms) to assess their individual and combined contribution to phenotype prediction. A baseline model including CYP3A5 alone was used as a reference to assess the incremental value of additional clinical and pharmacogenetic predictors.

The potential influence of concomitant medications known to affect CYP3A activity (e.g., corticosteroids and proton pump inhibitors) was also explored.

#### 2.2.5 Model evaluation

Models were compared using AIC. Model performance was assessed using likelihood-based pseudo-R^2^ statistics (McFadden) which estimates model goodness of fit by quantifying the improvement of the full model over a baseline model containing only the intercept.

Discriminative performance of the final model was assessed using Receiver Operating Characteristic (ROC) curve analysis and calculation of the area under the curve (AUC) with 95% confidence intervals. Classification metrics, including sensitivity, specificity, PPV, NPV, and overall accuracy, were derived from confusion-matrix analysis.

Internal validation of the final logistic regression model was performed using bootstrap resampling (200 iterations). For each bootstrap sample, the model was refitted and its discriminative performance was evaluated both in the bootstrap sample and in the original dataset to estimate optimism-corrected AUC.

## 3 Results

### 3.1 Patient characteristics

Baseline characteristics of the study population (n = 77), stratified by tacrolimus metabolism phenotype, are summarized in Table 1. The high- and low-metabolism groups included 39 and 38 patients, respectively. Patients with low-metabolism phenotype were significantly older and had a longer post-transplant period compared with high-metabolism patients. As expected by phenotype definition, low-metabolism patients showed higher tacrolimus trough concentrations and higher dose-normalized C0 and C0/D/W values, despite receiving lower absolute tacrolimus doses. The distribution of formulation type was similar between groups, although all patients with a 12-h dosing interval belonged to the high-metabolism group.

**TABLE 1:** Baseline demographic and clinical characteristics of the study population. Continuous variables are presented as mean ± SD, and categorical variables as counts and percentages.

Variable	High metabolism phenotype (n = 39)	Low metabolism phenotype (n = 38)	p-value
N	39	38	—
Age (years), mean (SD)	46.77 (12.46)	54.08 (10.98)	0.008
Gender, n (%)	​	​	1.000
Male	19 (48.7)	18 (47.4)	​
Female	20 (51.3)	20 (52.6)	​
Weight (kg), mean (SD)	71.86 (17.55)	63.95 (12.80)	0.027
BMI (kg/m^2^), mean (SD)	25.24 (4.18)	24.04 (4.01)	0.202
Ethnicity, n (%)	​	​	0.818
Caucasian	32 (82.1)	33 (86.8)	​
Latino	6 (15.4)	4 (10.5)	​
Black/African descent	1 (2.6)	1 (2.6)	​
Number of transplants, mean (SD)	1.26 (0.50)	1.39 (0.75)	0.344
Post-transplant period (years), mean (SD)	6.47 (4.78)	9.48 (6.31)	0.021
Tacrolimus trough level C0 (ng/mL), mean (SD)	5.81 (1.62)	7.52 (2.82)	0.002
Tacrolimus dose (mg), mean (SD)	5.45 (1.81)	2.49 (1.15)	<0.001
Dose-adjusted tacrolimus C0, mean (SD)	0.99 (0.34)	3.32 (1.27)	<0.001
Dose and weight- adjusted tacrolimus C0, mean (SD)	0.014 (0.003)	0.053 (0.02)	<0.001
Formulation, n (%)	​	​	0.809
Advagraf®	31 (79.5)	32 (84.2)	​
Envarsus®	8 (20.5)	6 (15.8)	​
Dosing interval (hours), n (%)	​	​	0.010
12 h	8 (20.5)	0 (0.0)	​
24 h	31 (79.5)	38 (100.0)	​
Hemoglobin (g/dL), mean (SD)	13.32 (1.82)	13.37 (1.38)	0.892
Hematocrit (%), mean (SD)	41.33 (5.53)	41.77 (4.21)	0.699
AST (U/L), mean (SD)	21.74 (18.77)	17.96 (6.08)	0.333
ALT (U/L), mean (SD)	26.81 (16.86)	25.31 (11.99)	0.710
Alkaline phosphatase (U/L), mean (SD)	82.04 (26.66)	87.58 (35.39)	0.522
GGT (U/L), mean (SD)	33.41 (27.12)	46.27 (51.69)	0.259

### 3.2 Distribution of genotypes by phenotype

All analyzed SNPs were in Hardy-Weinberg equilibrium (p > 0.05), indicating no significant deviation from expected genotype distributions in the study population. The distribution of ABCB1, CYP3A4, and CYP3A5 genotypes according to metabolism phenotype is presented in Table 2. No significant differences were observed in ABCB1 genotype frequencies between high- and low-metabolism groups. In contrast, CYP3A4 rs2242480 showed a trend towards different distributions between phenotypes. CYP3A5 rs776746 was proportionally enriched for expresser genotypes (*1 carriers) in the high-metabolism group and for the *3/*3 non-expresser genotype in the low-metabolism group (p = 0.001).

**TABLE 2:** Distribution of ABCB1, CYP3A4, and CYP3A5 genotypes according to tacrolimus metabolic phenotypes.

Gene and polymorphism	High metabolism phenotype	Low metabolism phenotype	p-value
ABCB1 rs1045642, n (%)	​	​	0.423
C/C	10 (25.6)	5 (13.2)	​
C/T	19 (48.7)	21 (55.3)	​
T/T	10 (25.6)	11 (28.9)	​
ABCB1 rs1128503, n (%)	​	​	0.728
C/C	17 (43.6)	18 (47.4)	​
T/C	17 (43.6)	14 (36.8)	​
T/T	5 (12.8)	5 (13.2)	​
ABCB1 rs2032582, n (%)	​	​	0.924
G/G	20 (51.3)	19 (50.0)	​
G/T	14 (35.9)	15 (39.5)	​
T/T	5 (12.8)	4 (10.5)	​
CYP3A4 rs2242480, n (%)	​	​	0.054
A/A	5 (12.8)	1 (2.6)	​
G/A	14 (35.9)	7 (18.4)	​
G/G	20 (51.3)	29 (76.3)	​
CYP3A5 rs776746 (*3), n (%)	​	​	0.001
A/A	2 (5.1)	1 (2.6)	​
A/G	17 (43.6)	3 (7.9)	​
G/G	20 (51.3)	34 (89.5)	​

### 3.3 CYP3A5 genotyping and phenotype prediction

To explore the relationship between pharmacokinetic phenotype and CYP3A5 genotype, a contingency table was constructed (Table 3). The CYP3A5*3/*3 haplotype (GG genotype) was used as the reference category, as it is the most prevalent variant in the Caucasian population and serves as the reference in CPIC guidelines, given that carriers usually initiate treatment with standard tacrolimus doses. In contrast, carriers of the *1/*1 and *1/*3 genotypes generally require dose adjustments.

**TABLE 3:** Distribution of CYP3A5 genotype according to tacrolimus phenotypic classification.

Tacrolimus C0/D/W phenotype	Expressers (*1 carriers)	Non-expressers (*3/*3)	Total
High metabolism phenotype	19	20	39
Low metabolism phenotype	4	34	38
Total	23	54	77

Among patients classified as high metabolism phenotype, 19 were CYP3A5 expressers (*1 carriers), resulting in a sensitivity of 48.7% (95% CI, 34–64). Conversely, 34 of 38 patients in the low metabolism phenotype group were non-expressers (*3/*3), corresponding to a specificity of 89.5% (95% CI, 76–96). The positive predictive value of the expresser genotype for identifying high metabolism phenotype was 82.6% (95% CI, 63–93), while the negative predictive value was 63% (95% CI, 50–75). Overall diagnostic accuracy of CYP3A5 genotype alone for phenotype classification was 68.8%.

### 3.4 Multivariate logistic regression integrating genetic and clinical predictors of tacrolimus pharmacokinetic phenotype

Multivariable analyses were performed in patients with complete clinical and genetic data (n = 75). Genetic scores were calculated for the five variants selected *a priori*: CYP3A5 rs776746, CYP3A4 rs2242480, and ABCB1 rs1045642, rs1128503, and rs2032582. Moderate multicollinearity was observed among ABCB1 polymorphisms, with variance inflation factors (VIF) of 4.76 for rs1128503 and 4.42 for rs2032582, compared with 2.20 for rs1045642. Accordingly, rs1045642 was retained for multivariable modeling, while all remaining predictors showed VIF values <2.

A baseline model including CYP3A5 score alone showed moderate explanatory capacity (AIC 92.53; McFadden pseudo-R^2^ 0.148).

A parsimonious multivariable model including age, post-transplant period, formulation type, and CYP3A5 score improved model fit substantially (AIC 87.32; McFadden pseudo-R^2^ 0.256). In this model, age (OR 1.05, 95% CI 1.00–1.11; p = 0.038), post-transplant period (OR 1.10, 95% CI 1.00–1.22; p = 0.050), and CYP3A5 score (OR 12.6, 95% CI 3.18–88.05; p = 0.002) were associated with phenotype classification, while formulation showed a borderline association (OR 6.37, 95% CI 1.07–60.69; p = 0.063) (Table 4).

**TABLE 4:** Multivariate logistic regression model integrating CYP3A5 rs776746 genetic score and clinical covariates.

Variable	β (Estimate)	OR	IC 95%	p-value
Age	0.005	1.05	1.00–1.11	0.038
Post-transplant period	0.096	1.10	1.00–1.22	0.050
Formulation (Envarsus® vs. Advagraf®)	1.851	6.37	1.07–60.69	0.063
CYP3A5 rs776746 score	2.537	12.6	3.18–88.05	0.002

Addition of CYP3A4 rs2242480 did not improve model performance (AIC 88.85; McFadden pseudo-R^2^ 0.260) and was not significantly associated with phenotype (p = 0.497) (Supplementary Table S1). By contrast, inclusion of ABCB1 rs1045642 resulted in a modest improvement in model fit (AIC 85.19; McFadden pseudo-R^2^ 0.295). In this extended model, CYP3A5 score remained the strongest predictor (OR 17.6, 95% CI 4.01–135.93; p < 0.001), while ABCB1 rs1045642 showed a borderline association (OR 2.54, 95% CI 1.03–7.11; p = 0.054) (Table 5).

**TABLE 5:** Extended multivariate logistic regression model integrating CYP3A5 rs776746 and ABCB1 rs1045642 genetic scores and clinical covariates.

Variable	β (Estimate)	OR	IC 95%	p-value
Age	0.052	1.05	1.00–1.11	0.038
Post-transplant period	0.123	1.13	1.02–1.27	0.023
Formulation (Envarsus® vs. Advagraf®)	2.582	13.2	1.79 –164.73	0.022
CYP3A5 rs776746 score	2.871	17.6	4.01–135.93	<0.001
ABCB1 rs1045642 score	0.934	2.54	1.03–7.11	0.054

**FIGURE 1:**
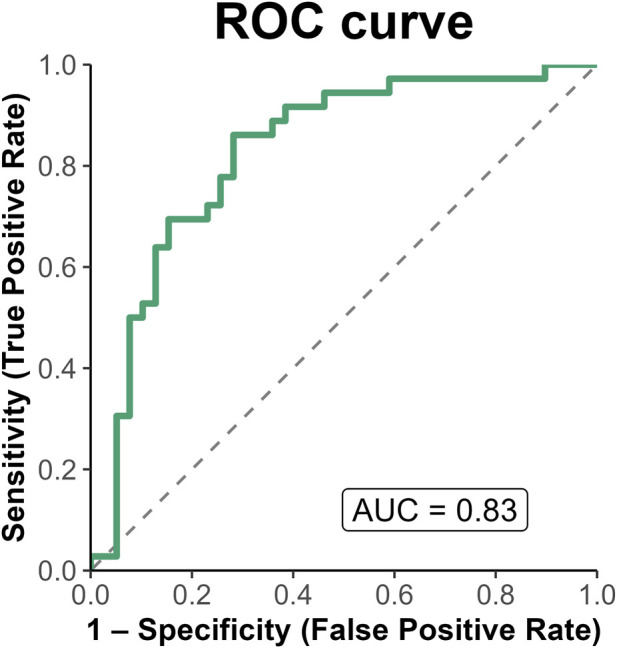
ROC curve of the parsimonious multivariable logistic regression model. AUC = 0.83 (95% CI 0.73–0.92).

Given the limited incremental improvement in model performance (ΔAIC = 2.13) indicating no meaningful advantage over the simpler model (Burnham and Anderson, 2004) and the borderline statistical significance of ABCB1 rs1045642, the parsimonious model was retained as the primary model for subsequent evaluation of goodness-of-fit, discrimination, and internal validation. The parsimonious model demonstrated good discriminative performance, with an apparent AUC of 0.83 (95% CI 0.73–0.92) and an optimism-corrected AUC of 0.80 after bootstrap internal validation (200 iterations), indicating limited overfitting (Figure 1).

No association was observed between concomitant medications affecting tacrolimus exposure phenotype.

## 4 Discussion

Tacrolimus dosing remains a major challenge in the clinical management of kidney transplant recipients. The drug displays substantial interindividual pharmacokinetic variability, which complicates dose individualization and increases the risk of both underexposure, leading to rejection, and overexposure, which may result in toxicity. Strategies capable of improving dose precision are therefore of considerable clinical interest. The tacrolimus trough concentration-to-dose ratio (C0/D) and its weight-normalized variant (C0/D/W) have emerged as informative phenotypic markers of tacrolimus metabolism and exposure, with well-established clinical relevance in kidney transplantation. Multiple studies conducted across heterogeneous transplant cohorts consistently show that a low C0/D ratio, reflecting a fast-metabolizer phenotype, is associated with lower estimated glomerular filtration rate, accelerated long-term decline in graft function, increased interstitial fibrosis and tubular atrophy, higher rates of calcineurin inhibitor–related nephrotoxicity, and poorer death-censored graft survival (Schütte-Nütgen et al., 2019; Thölking et al., 2019; Jouve et al., 2020; Van Gelder et al., 2020).

The clinical relevance of tacrolimus metabolic phenotypes aligns with current pharmacogenetic evidence. Major clinical practice guidelines, including those from the Clinical Pharmacogenetics Implementation Consortium and the Dutch Pharmacogenetics Working Group, consistently recognize CYP3A5 as a principal determinant of tacrolimus disposition. Among the genetic variants studied, CYP3A5*3 (rs776746) remains the most robust and reproducibly validated predictor of tacrolimus clearance, with CYP3A5 expressers generally requiring higher tacrolimus doses to achieve target trough concentrations.

In this context, we evaluated the ability of CYP3A5 rs776746 genotype to predict the tacrolimus C0/D/W-defined metabolism phenotype. In our cohort of stable adult kidney transplant recipients treated with prolonged-release tacrolimus, CYP3A5 genotype demonstrated high specificity but limited sensitivity for phenotype classification. Nearly 90% of low-metabolism patients were correctly identified as non-expressers, but more than half of high-metabolism patients were misclassified, highlighting that CYP3A5 alone does not fully capture the determinants of tacrolimus exposure.

Although additional polymorphisms in genes such as CYP3A4 and ABCB1 have been proposed as modifiers of tacrolimus pharmacokinetics, their contributions are less consistent across studies and typically modest in magnitude. Importantly, genetic determinants interact with several clinical factors, most notably age, hematocrit, hemoglobin, and renal function, which have all been identified as significant contributors to the extensive variability observed in tacrolimus pharmacokinetics at some studies (Ana Luisa and Manuel Alejandro, 2011; Stratta et al., 2012; Birdwell et al., 2015).

In this context and to further explore this variability, we assessed the contribution of clinical and additional pharmacogenetic factors. In univariable analyses, age and post-transplant period were significantly associated with phenotype classification, whereas hematological parameters and renal function were not. Although hematocrit is known to influence whole-blood tacrolimus concentrations through erythrocyte binding, its clinical impact may be attenuated in stable transplant populations with limited variability (Størset et al., 2014). Similarly, tacrolimus clearance is primarily determined by hepatic metabolism and is largely independent of glomerular filtration rate, although indirect relationships with renal function cannot be completely excluded (Staatz and Tett, 2004).

Regarding pharmacogenetic variants, CYP3A4 rs2242480 showed a trend towards association with tacrolimus metabolism phenotype in univariable analysis, whereas ABCB1 polymorphisms were not significantly associated. In contrast, CYP3A5 rs776746 showed the strongest and most consistent association, confirming its central role in tacrolimus disposition.

To better capture the multifactorial nature of tacrolimus pharmacokinetics, we constructed genotype-based exposure scores for variants previously implicated in tacrolimus metabolism and transport and evaluated their combined effect with relevant clinical covariates. In this context, a baseline model including CYP3A5 alone showed moderate explanatory capacity. However, the addition of key clinical variables, particularly age and post-transplant period, resulted in a substantial improvement in model performance. This was quantitatively reflected by a marked reduction in AIC and a clear increase in McFadden’s pseudo-R^2^, indicating improved goodness-of-fit and explanatory power.

Although the magnitude of these effects was modest, both variables showed consistent associations with phenotype classification. Increasing age was associated with a higher probability of low-metabolism phenotype (OR 1.05; 95% CI 1.00–1.11; p = 0.038), which is biologically plausible given age-related reductions in hepatic blood flow and CYP3A activity, leading to decreased tacrolimus clearance and higher dose-corrected exposure (Andrews et al., 2019). Similarly, a longer post-transplant period showed a borderline association (OR 1.10; 95% CI 1.00–1.22; p = 0.050), consistent with the well-described progressive increase in tacrolimus exposure over time after transplantation (Kuypers et al., 2004).

Tacrolimus formulation also showed a trend towards association with the low-metabolism phenotype (OR 6.37; 95% CI 1.07–60.69; p = 0.063). This observation is consistent with the known pharmacokinetic properties of LCPT/MeltDose tacrolimus, which provides increased bioavailability and a more stable concentration–time profile compared with other prolonged-release formulations (Tremblay et al., 2017). The wide confidence interval likely reflects limited statistical power due to the small number of patients receiving Envarsus® in this cohort.

The inclusion of additional pharmacogenetic variants yielded limited incremental benefit. The addition of CYP3A4 rs2242480 did not improve model performance and was not significantly associated with phenotype after adjustment, in line with previous studies reporting modest and inconsistent effects of CYP3A4 polymorphisms (Birdwell et al., 2015; Brunet et al., 2019). In contrast, inclusion of ABCB1 rs1045642 resulted in a limited improvement in model fit, as reflected by a slight reduction in AIC, although its association with phenotype remained borderline and did not reach statistical significance. This finding is consistent with prior evidence suggesting that ABCB1 polymorphisms are not decisive determinants of tacrolimus pharmacokinetics and show inconsistent effects across studies (Yamada et al., 2020). Taken together, these results support a limited incremental value of ABCB1 beyond CYP3A5 in the prediction of tacrolimus exposure.

The final model, integrating CYP3A5 rs776746 with key clinical covariates, provided a robust and clinically interpretable framework for phenotype prediction. This model demonstrated good discriminative performance, with an apparent AUC of 0.83 and an optimism-corrected AUC of 0.80 after bootstrap internal validation, indicating limited overfitting despite the relatively small sample size.

Overall, our findings support the concept that tacrolimus pharmacokinetics is primarily driven by CYP3A5 genotype, although this alone does not fully explain interindividual variability in exposure. The incorporation of key clinical variables significantly improves predictive performance, whereas additional pharmacogenetic variants provide only modest incremental contributions. These results are consistent with current consensus recommendations emphasizing CYP3A5 as the primary pharmacogenetic determinant of tacrolimus exposure (Brunet et al., 2019). Future studies in larger and more diverse cohorts are warranted to further clarify the contribution of secondary pharmacogenetic markers and to validate integrative prediction models (Cheng et al., 2022; He et al., 2022; Min et al., 2025).

### 4.1 Limitations

This study has several limitations that should be considered when interpreting the findings. First, its retrospective single-centre design and modest sample size may limit generalizability and increase the risk of overfitting, despite the use of bootstrap internal validation. Second, the analysis focused on extreme C0/D/W tertiles, which enhances phenotype contrast but may not fully represent intermediate phenotypes. This approach inevitably reduces the effective sample size and limits the representativeness of the full population of kidney transplant recipients. The strategy was deliberately adopted to maximize phenotypic contrast between groups and to improve the detectability of underlying biological determinants of tacrolimus exposure. Intermediate phenotypes frequently represent a heterogeneous mixture of patients with overlapping pharmacokinetic characteristics, potentially diluting associations with pharmacogenetic or clinical predictors. Nevertheless, this design choice means that the current model does not directly address patients with intermediate exposure profiles, and future studies including the full spectrum of phenotypes will be necessary to assess the applicability of these findings across the broader transplant population. Third, we did not systematically evaluate the impact of intrapatient variability, or adherence, both of which can influence tacrolimus exposure. Fourth, although the genetic variants were selected a priori based on their functional relevance to tacrolimus pharmacokinetics, the potential for false-positive findings cannot be excluded and the results therefore require external validation in larger cohorts. A further limitation of this study is the lack of donor genotyping. Emerging evidence suggests that CYP3A5 is expressed not only in hepatic tissue but also in renal tissue, and that donor CYP3A5 genotype may influence drug metabolism and exposure in kidney transplant recipients (Warzyszyńska et al., 2022). Therefore, the absence of donor genetic data may have limited our ability to fully capture interindividual variability in pharmacokinetics. Finally, we did not directly evaluate clinical outcomes such as acute rejection, nephrotoxicity, or long-term graft survival in relation to the predicted phenotype. Despite these limitations, the proposed score may help identify patients with different tacrolimus exposure profiles. In the future, this type of pharmacogenetic information could be integrated with clinical variables into Model-Informed Precision Dosing (MIPD) approaches to support individualized tacrolimus dosing in kidney transplant recipients.

## 5 Conclusion

In conclusion, our study confirms that CYP3A5 genotype is the primary determinant of tacrolimus C0/D/W-based metabolism phenotype in stable adult kidney transplant recipients treated with prolonged-release formulations. However, its use alone provides high specificity but limited sensitivity for phenotype discrimination. Incorporation of key clinical variables, particularly age, formulation type, and time since transplantation, improved predictive performance and provided a more comprehensive characterization of tacrolimus exposure variability. Additional pharmacogenetic markers showed only modest incremental value and should be interpreted with caution.

These findings support a combined genetic-clinical approach as a pragmatic framework to better capture interindividual variability in tacrolimus pharmacokinetics and to inform dose individualization in clinical practice. Nevertheless, external validation in larger prospective cohorts is required to confirm the robustness and clinical utility of this model and to assess its impact on transplant outcomes.

## Data Availability

The raw data supporting the conclusions of this article will be made available by the authors upon request.
