# Supplementary material for: Integrating pharmacogenetic and clinical factors to predict the C0/D/W-based tacrolimus phenotype in kidney transplantation
Source: Front Pharmacol. 2026 May 15;17:1772820. doi: 10.3389/fphar.2026.1772820 (PMC13219021; doi:10.3389/fphar.2026.1772820)
Supplement: Supplementary file 1 [file Table1.docx]

| Variable | β (Estimate) | OR | IC 95% | p-value |
| --- | --- | --- | --- | --- |
| Age | 0.053 | 1.06 | 1.01–1.11 | 0.032 |
| Post-transplant period | 0.093 | 1.10 | 1.00–1.22 | 0.060 |
| Formulation (Envarsus® vs. Advagraf®) | 1.785 | 5.96 | 0.99 –56.6 | 0.074 |
| CYP3A5 rs776746 score | 3.149 | 23.3 | 2.55–372.04 | 0.011 |
| CYP3A4 rs2242480 score | -0.592 | 0.553 | 0.09–3.03 | 0.497 |

**Table S1. Extended multivariate logistic regression model integrating CYP3A5 rs776746 and CYP3A4 rs2242480 genetic scores and clinical covariates**
